# Convoys, Network Advantaage, and Mental Health in Older Adulthood

**DOI:** 10.1093/geroni/igaf122.1864

**Published:** 2025-12-31

**Authors:** James Iveniuk, I’deyah Ricketts

**Affiliations:** National Opinion Research Center at The University of Chicago, Scarborough, Ontario, Canada; NORC at the University of Chicago, Chicago, Illinois, United States

## Abstract

The convoy model is one of the most influential theories in gerontology of how social network change shapes psychological wellbeing over the life course. However, it is rare that researchers have the data to observe changes in social networks by emotional closeness to network members – especially in nationally-representative data. In this paper, we draw on three waves of the National Social Life Health and Aging Project, describe social network change at different levels of emotional closeness to the respondent, and then explore associations between network change and levels of depression. We find that a) Black respondents are far more likely to experience turnover (gains and losses) among their closest confidants, b) women are less likely to experience turnover in not-so-close confidants, and c) losing not-so-close confidants was associated with greater depression, and d) gaining new extremely close confidants was associated with lower levels of depression. We close with implications for dialogue between convoy theories, and theories of network advantage.

